# Supplementary material for: Diverse crystal size effects in covalent organic frameworks
Source: Nat Commun. 2020 Nov 30;11:6128. doi: 10.1038/s41467-020-19858-8 (PMC7705719; doi:10.1038/s41467-020-19858-8)
Supplement: Supplementary file 1 — Supplementary Information [file 41467_2020_19858_MOESM1_ESM.pdf]

[*Supplementary Information*]

Diverse Crystal Size Effects in Covalent Organic Frameworks

Tianqiong Ma[1,2], Lei Wei[3], Lin Liang[2], Shawn Yin[4], Le Xu[1], Jing Niu[2], Huadong Xue[2], Xiaoge Wang[1], Junliang Sun[1,*], Yue-Biao Zhang[3,*], Wei Wang[2,*]

[1] College of Chemistry and Molecular Engineering, Beijing National Laboratory for Molecular Sciences (BNLMS), Peking University, Beijing 100871, PR China.

[2] State Key Laboratory of Applied Organic Chemistry, College of Chemistry and Chemical Engineering, Lanzhou University, Lanzhou, Gansu 730000, PR China.

[3] School of Physical Science and Technology, ShanghaiTech University, Shanghai 201210, PR China.

[4] Drug Product Development Bristol-Myers Squibb Co., One Squibb Drive, New Brunswick, NJ 08903, USA.

*E-mail: junliang.sun@pku.edu.cn

zhangyb@shanghaitech.edu.cn

wang_wei@lzu.edu.cn

Supplementary Methods

Materials. Aniline (AR, ≥ 99.5%) was purchased from Chengdu Chron Chemicals Co. Terephthalaldehyde (BDA), 1,4-dioxane (AR, ≥ 99.5%) and glacial acetic acid (AR, ≥ 99.5%) were purchased from Tianjin Guangfu Fine Chemical Research Institution. All regents and solvents were used without further purification unless otherwise specified. tetrakis(4-formylphenyl)silane (TFS) and tetrakis(4-aminophenyl)methane (TAM) were synthesized according to the reported procedures.[1,2]

Powder X-ray diffraction (PXRD). The PXRD data were collected on PANalytical X' Pert diffractometer and on PANalytical Empyrean diffractometer with the Cu Kα radiation of λ = 1.5418 Å at 40 kV and 40 mA. Prior to analysis, some of the samples were ground, and then mounted on flat sample holders or sealed in capillary. The samples were measured with the Bragg angle (2θ) ranged from 3.0° to 35.0° or 5.0° to 50.0° with a step size of 0.013° and a scan time of 2 s per step.

Solid-state nuclear magnetic resonance (SSNMR). All the SSNMR experiments were performed with magic angle spinning (MAS) on a Bruker Avance II 400 MHz wide-bore solid-state NMR spectrometer at a magnetic field of 9.4 T. ^{13}C MAS NMR data were acquired at the Larmor frequency (ν_0) of 100.6 MHz and ^{29}Si MAS NMR data were acquired at ν_0 of 79.5 MHz. Both the ^{13}C and ^{29}Si chemical shifts were referenced to tetramethylsilane (TMS) at 0 ppm (δ_{iso}). All the ^{13}C and ^{29}Si experiments were carried out on a standard 4 mm double-resonance probe with the sample spinning rate of 10 kHz. For ^{13}C high power decoupling (HPDEC) MAS measurement, a ^{13}C π/2 pulse length of 4.0 μs and a pulse delay of 5 s were applied; a two-pulse phase-modulation (TPPM) sequence was used for the proton decoupling with a decoupling frequency of 62.5 kHz. The ^{13}C cross-polarization (CP) MAS experiments were carried out with a ^{1}H π/2 pulse length of 3.2 μs, a contact time of 3 ms, a pulse delay of 3 s, and a TPPM decoupling frequency of 78.1 kHz. The ^{29}Si CP/MAS spectra were acquired with a ^{1}H π/2 pulse length of 4.0 μs, a contact time of 6.5 ms, a pulse delay of 3 s, and a TPPM decoupling frequency of 62.5 kHz.

^{129}Xe NMR. Standard Xe gas (chemical purity of 99.999%, Guangzhou Shiyuan Gases) with the natural abundance (26.44%) of ^{129}Xe was used for all the ^{129}Xe adsorption experiments. ^{129}Xe NMR spectra were recorded on the Bruker Avance II 400 MHz wide-bore solid-state NMR spectrometer mentioned above. The samples were fused in NMR glass tubes and measured without MAS. Before the measurement, an NMR glass tube was charged with the COFs samples and degassed under vacuum at 120 °C for 24 h. Then Xe gas with the pressure of 10 mbar was condensed into the tube within a liquid N_2 bath. Finally, the NMR glass tube was flame sealed and subjected to the ^{129}Xe measurement at ambient temperature with the Larmor frequency (ν_0) of 111.2 MHz. A ^{129}Xe $\pi/2$ pulse length of 4.5 μs and a pulse delay of 2 s were used. The ^{129}Xe chemical shifts were referenced to the chemical shift of Xe gas extrapolated to zero pressure.

Gas adsorption-desorption experiments. The N_2 adsorption-desorption experiments of LZU-111 were conducted on a Micromeritics ASAP 2020 Surface Area and Porosimetry Analyzer. The samples were degassed at 120 °C for 12 h before the measurements. N_2 isotherms were generated at 77 K by incremental exposure to high purity nitrogen up to 1 atm. Data analyses were conducted with the software ASAP 2020 V4.01. The Ar adsorption-desorption experiments of LZU-111 and the gas adsorption-desorption experiments of COF-300 were conducted on a Quantachrome Autosorb-iQ gas adsorption analyzer. The samples were degassed at 120 °C for 12 h before the measurements. N_2 isotherms were generated at 77 K by incremental exposure to high purity nitrogen up to 1 atm. Ar isotherms were generated at 87 K by incremental exposure to high purity argon up to 1 atm. CO_2 isotherms were generated at 195 K by incremental exposure to high purity carbon dioxide up to 1 atm and dry ice-methanol bath was used for the temperature controlled at 195 K. The organic vapors (tetrahydrofuran, 1,4-dioxane, ethyl alcohol and isopropanol) adsorption isotherms were collected using MicrotracBELSopr-Aqua3 adsorption apparatus with a water circulator bath. Water bath with circulator were used for temperatures controlled at 283 and 298 K. Anhydrous solvents were used for vapor adsorption,

which degassed at least five times before isotherm collection.

Other characterization methods. The scanning electron microscopy (SEM) images were obtained on a Hitachi S-4800 field emission scanning electron microscope at the accelerating voltages of 5.0 to 10.0 kV. The Fourier transform infrared (FT-IR) spectra were collected on a Bruker ALPHA FT-IR Spectrometer equipped with ALPHA's Platinum ATR single reflection diamond ATR module. The thermogravimetric (TG) curves were recorded on a TA SDT Q600 simultaneous thermal analyzer, and the samples were heated with a heating rate of 10.0 °C/min from ambient temperature to 800 °C in nitrogen atmosphere.

Supplementary Figures

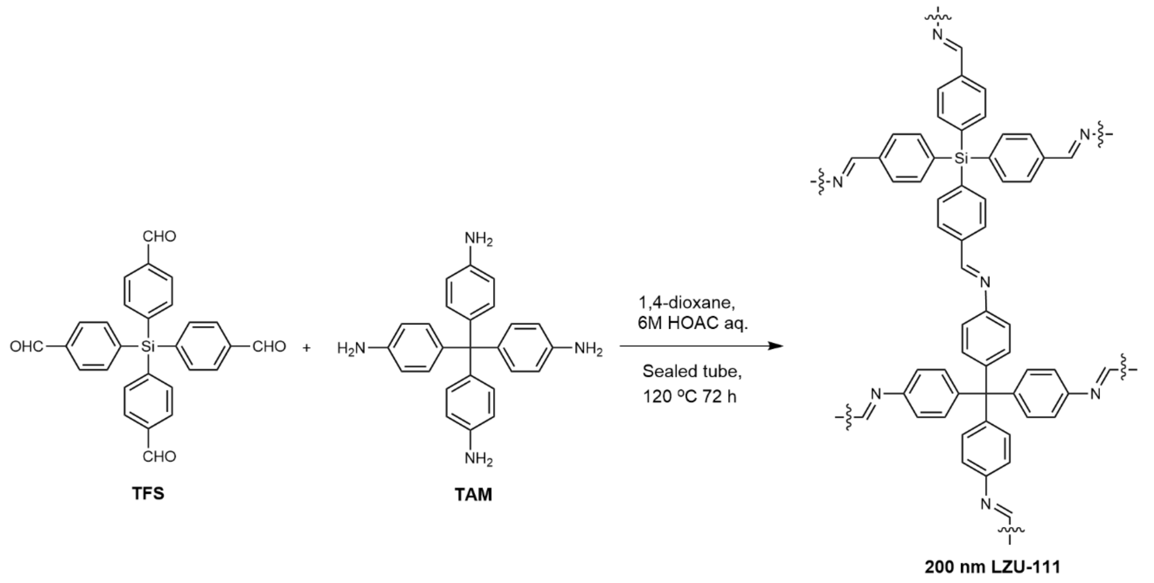

Supplementary Fig. 1 Crystallization of polycrystalline LZU-111 with crystal size of ~200 nm. The conventional solvothermal method for the synthesis of imine COFs[3] resulted in the formation of LZU-111 as nano-sized polycrystals. The synthetic conditions have been systematically optimized, and the typical procedure is listed as follows. A 10 mL glass tube was charged with TFS (22.4 mg, 0.05 mmol), TAM (19.0 mg, 0.05 mmol), and 0.6 mL of 1,4-dioxane. Then 0.2 mL of aqueous acetic acid (6.0 M) was added into the solution. The tube was flash frozen in a liquid N_2 bath, evacuated to vacuum and flame sealed. The fused tube was then heated at 120 °C for 72 h, during which a light-yellow solid was formed. The crude product was isolated by centrifugation and activated by a series of procedures such as Soxhlet extraction in 1,4-dioxane for 24 h, dried at ambient temperature for 12 h, and further dried at 120 °C for 12 h to afford a light-yellow powder. Yield: 75.8% (28.6 mg). The obtained material was characterized as the nano-sized crystals of LZU-111, which was insoluble in boiling water and common organic solvents such as acetone, methanol, tetrahydrofuran, and *N*, *N*-dimethylformamide.

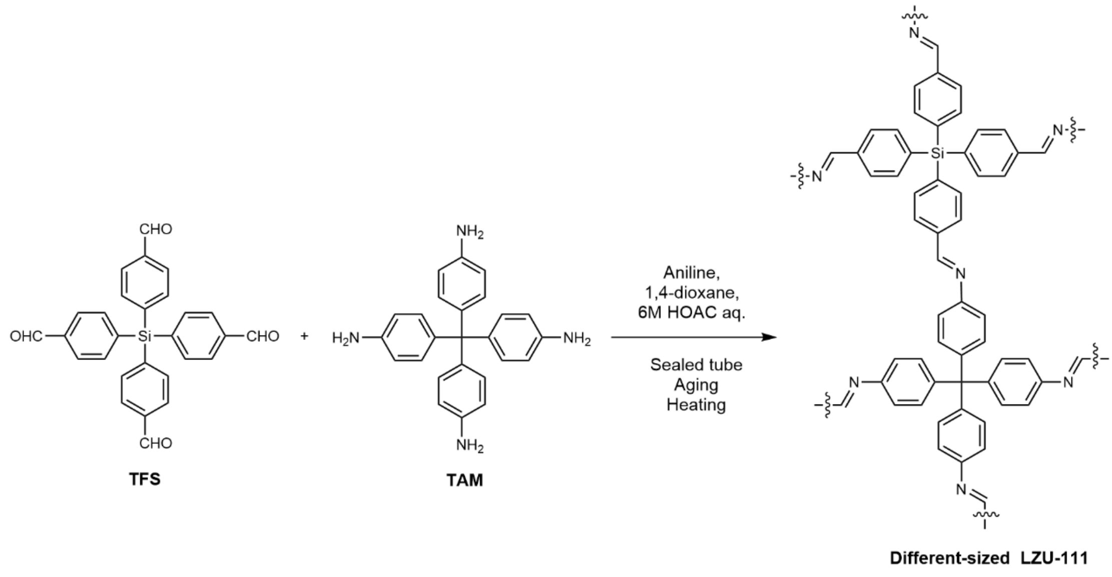

Supplementary Fig. 2 Crystallization of single-crystalline LZU-111 with different crystal sizes (from ~1 to ~30 μm). The key strategy for controllably synthesizing of different-sized LZU-111 is the addition of aniline as modulator with different amounts, which offered different degrees of inhibition for nucleation. The typical procedure is listed as follows: The 10 mL glass tubes were charged with TFS (22.4 mg, 0.05 mmol), aniline (0.07–0.27 mL, 15–60 equiv.) and 0.5 mL of 1,4-dioxane, then 0.2 mL of aqueous acetic acid (6 M) was added to the solutions. TAM (19.0 mg, 0.05 mmol) dissolved in 0.5 mL 1,4-dioxane was then added. Mixtures with different turbidity were obtained, which were sealed in tubes and left to stand at ambient temperature for 0.5 h–3 d and warmed at 40 °C for 1–3 d as an aging process, and further heated at 120 °C for 1–3 d. Then the different-sized single crystals of LZU-111 crystallized out, and the crystal sizes ranged from ~ 1 μm to ~ 30 μm. The more detailed information was summarized in Supplementary Table 1. Further activation for these samples were carried out as procedures for activation of 200 nm-sized LZU-111 to afford yellow crystals. If necessary, the crystals larger than 1 μm were further dried under vacuum at 120 °C for another 12 h to remove the guest molecules thoroughly. The phase purity of these samples was then verified by PXRD and SEM. The crystal-size-controlled synthesis of LZU-111 also confirms the versatility of synthetic strategy reported in our previous work.[4]

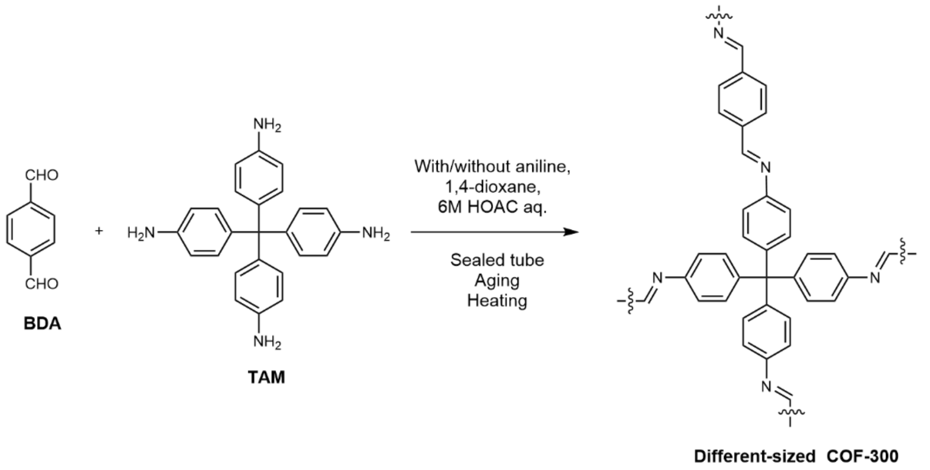

Supplementary Fig. 3 Crystallization of single-crystalline COF-300 with different crystal sizes (from ~500 nm to ~30 *μ*m). The crystallization of single-crystalline COF-300 (**dia-c7**) with the average crystal size from ~500 nm to ~30 *μ*m was reported in our previous works[4,5] and some reaction conditions were optimized accordingly here, see the more detailed information summarized in Supplementary Table 2.

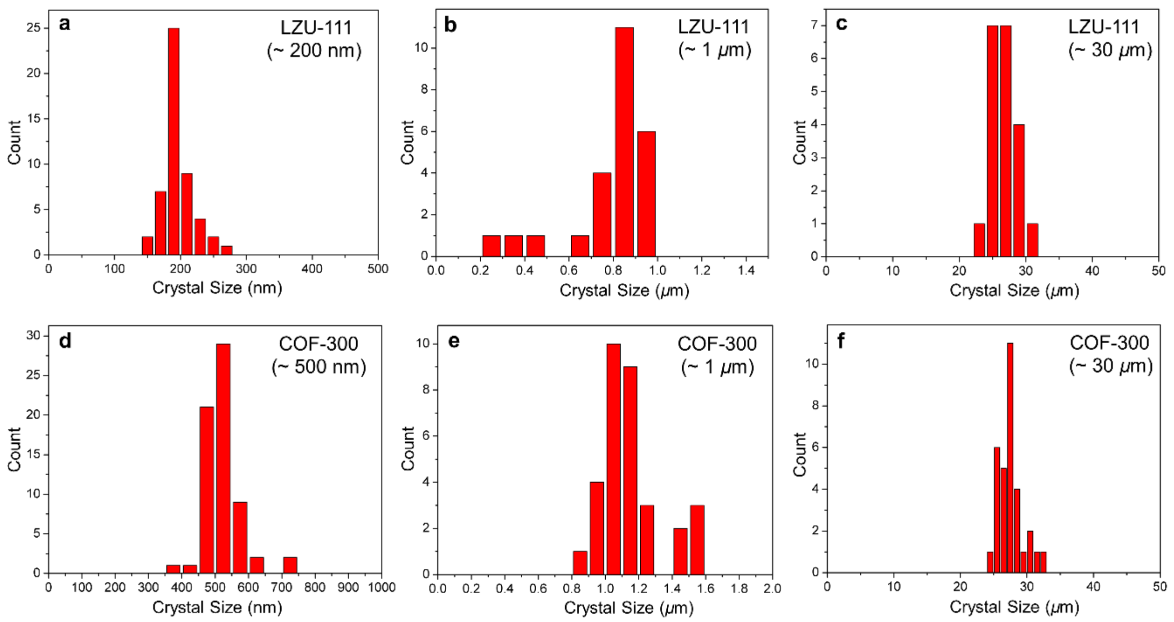

Supplementary Fig. 4 Statistical crystal size distributions (corresponding to the SEM information in Fig. 2 in main text). The statistics was implemented and analyzed with *Image J*. **a.** The size of nano LZU-111 crystals distributed from 150 to 270 nm and centered at 200 nm, represented by 200 nm-sized LZU-111. **b.** The size of LZU-111 crystals distributed mainly from 0.7 to 1.0 μm, represented by 1 μm -sized LZU-111. **c.** The size of micro-sized LZU-111 crystals distributed from 24 to 31 μm, represented by 30 μm-sized LZU-111. **d.** The size of nano COF-300 crystals mainly distributed from 450 to 600 nm and centered at 500 nm, represented by 500 nm-sized COF-300. **e.** The size of COF-300 crystals distributed mainly from 0.8 to 1.2 μm and centered at 1.0 μm, represented by 1 μm-sized COF-300. **f.** The size of micro-sized COF-300 crystals distributed from 24 to 33 μm, represented by 30 μm-sized COF-300.

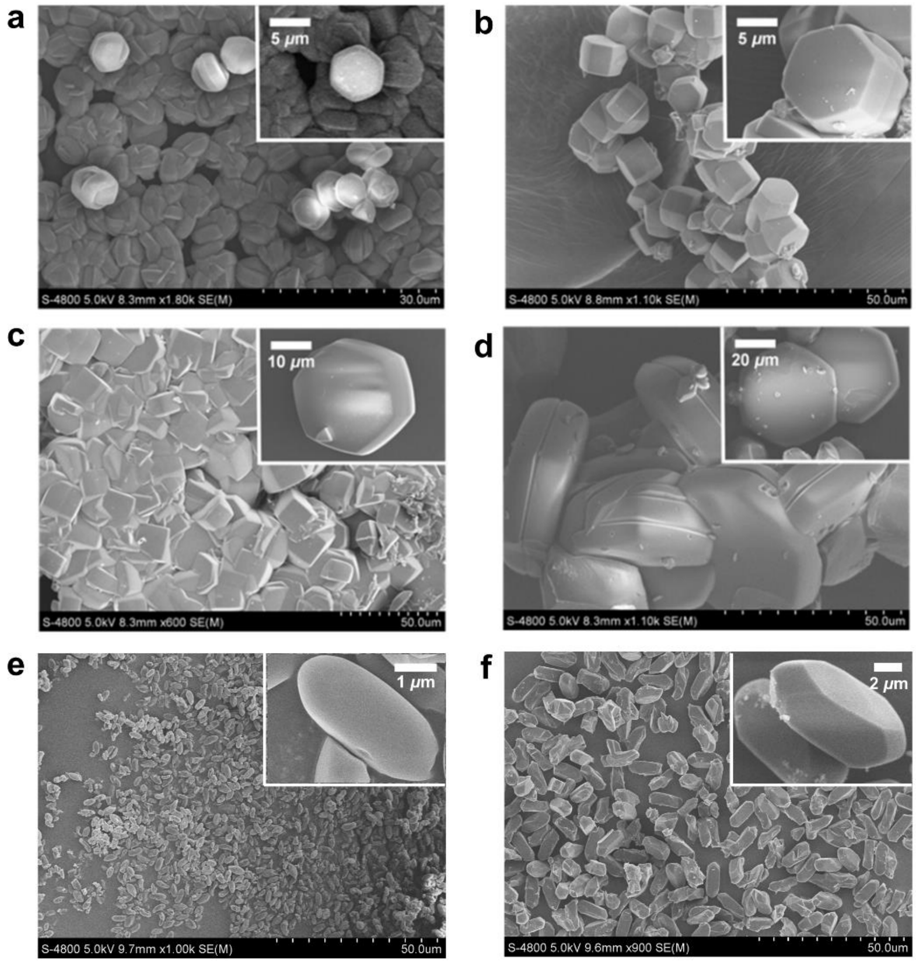

Supplementary Fig. 5 SEM images of different-sized LZU-111 and COF-300 crystals. a–d, LZU-111 crystals with average sizes of ~5 μm, ~10 μm, ~25 μm, and ~45 μm, respectively. **e–f,** COF-300 crystals with average sizes of ~4 μm and ~10 μm, which were used in the sorption experiments in Supplementary Fig. 14.

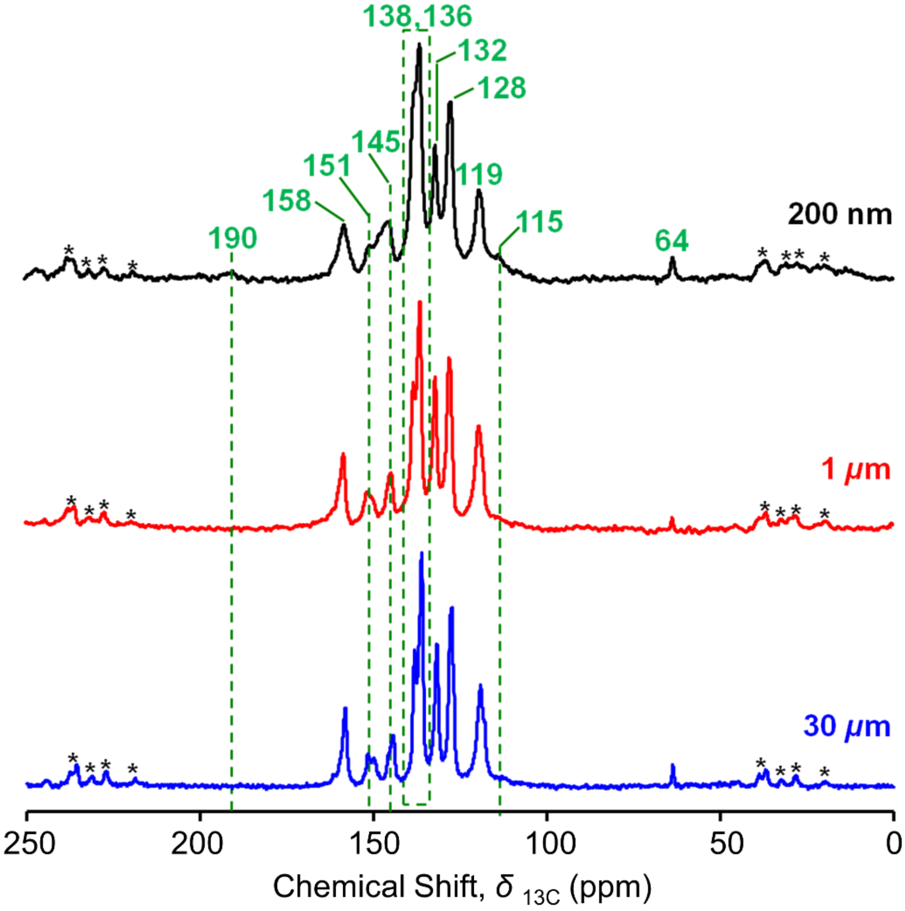

Supplementary Fig. 6 ^{13}C HPDEC/MAS spectra of different-sized LZU-111 (200 nm-sized, black; 1 μm-sized, red; 30 μm-sized, blue). Asterisks denote spinning sidebands. Similar to ^{13}C CP/MAS spectra of different-sized LZU-111 shown in Fig. 3b, the full width at half maximum (FWHM) of the ^{13}C NMR signals are decreased along with the increasing crystal size (from 200 nm to 30 μm). For example, the FWHM of the peak at 158 ppm is 346 Hz for 200 nm-sized crystals, 211 Hz for 1 μm-sized crystals, and 180 Hz for 30 μm-sized crystals. These results indicate that the crystallinity is significantly improved along with the increasing crystal size. Besides, the minor signals at 190 (corresponding to the terminal −CHO group) and 114 ppm (related to the terminal $-NH_2$ group) are the indictors for evaluating the possible defects in LZU-111 (the detailed assignments for the ^{13}C NMR signals of LZU-111 have been shown in Fig. 3b and also been reported in literature[4]). These signals could be observed obviously for the 200 nm-sized crystals but are nearly absent for the 30 μm-sized crystals, first because with the increasing of the crystal size, the

condensation reaction occurs more completely, thus pore integrity of LZU-111 is significantly improved along with the increased crystal size and crystallinity. Besides, part of the residual –CHO in 1 μm- and 30 μm-sized crystals could react with the excess amount of aniline in reversible imine condensation/exchange reactions.

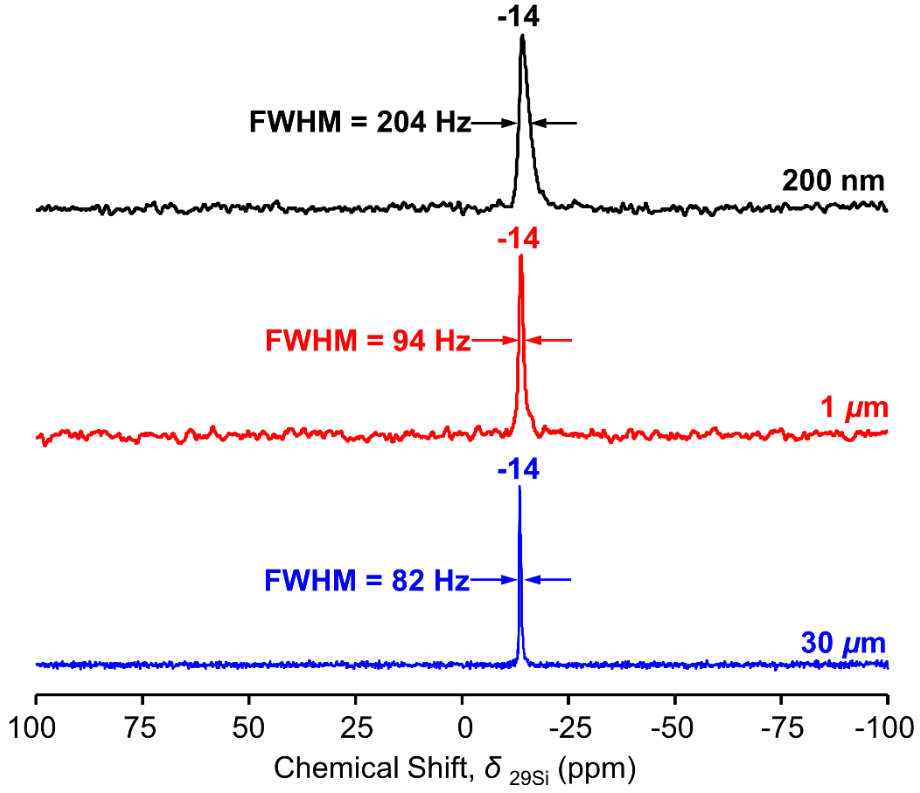

Supplementary Fig. 7 ^{29}Si CP/MAS spectra of LZU-111 with different crystal sizes (200 nm-sized, black; 1 μm-sized, red; 30 μm-sized, blue). The FWHM of the ^{29}Si NMR signal at −14 ppm is decreased (from 204 to 82 Hz) along with the increasing crystal size (from 200 nm to 30 μm). These results also demonstrate that the crystallinity of LZU-111 is significantly improved along with the increasing crystal size.

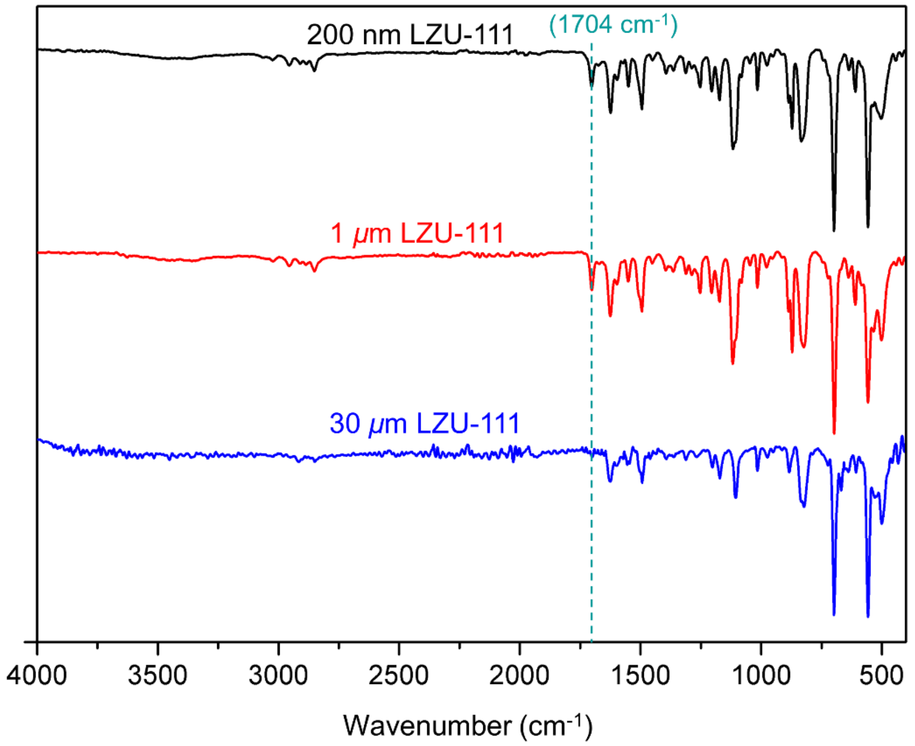

Supplementary Fig. 8 FT-IR spectra of different-sized LZU-111 (200 nm-sized, black; 1 *μ*m-sized, red; 30 *μ*m-sized, blue). The transmittance along the Y−axis is normalized for comparison. The band for the terminal −CHO group at 1704 cm^{-1} could be observed for the 200 nm- and 1 *μ*m-sized crystals and is almost absent in the FT-IR spectrum of the 30 *μ*m-sized crystals. It implies the 30 *μ*m-sized crystals contain very few defects such as the terminal −CHO group. These results indicate again that the pore integrity of LZU-111 was significantly improved along with the increased crystal size, which is consistent with the results from SSNMR analyses.

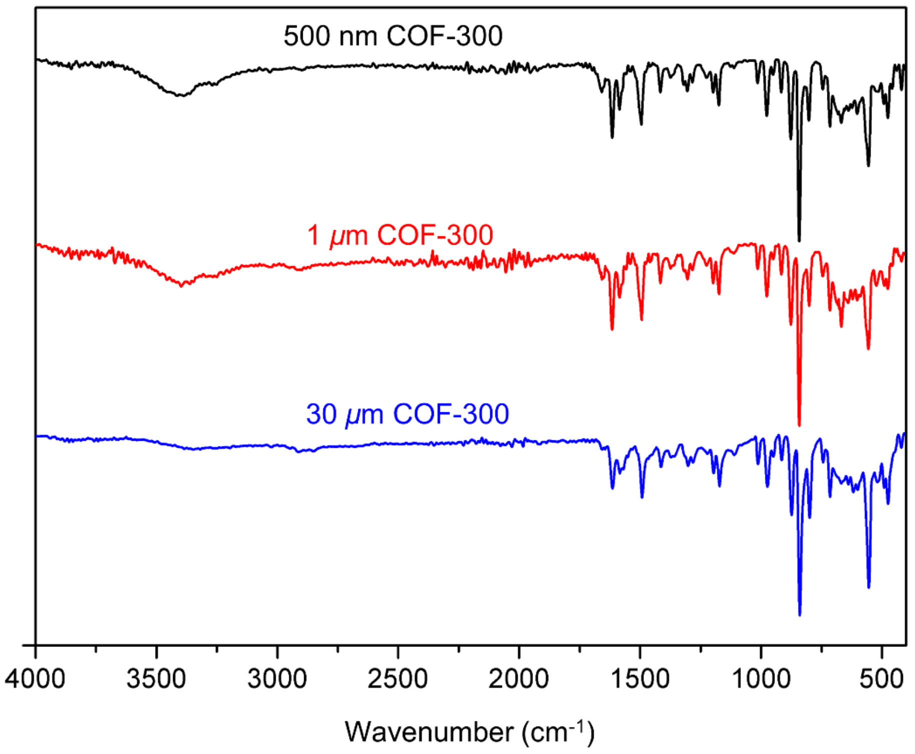

Supplementary Fig. 9 FT-IR spectra of COF-300 with different crystal sizes (500 nm-sized, black; 1 μm-sized, red; 30 μm-sized, blue). The transmittance along the Y−axis is normalized for comparison. The band for the –CHO group in BDA (at 1693 cm^{-1})[5] are absent in all the FT-IR spectra of the different-sized COF-300 crystals, which means all these samples contain few defects such as the terminal –CHO group. These results indicate further that the pore integrities of three-sized COF-300 crystals are nearly the same. This is consistent with the results from PXRD and SSNMR analyses which show in the main text (Figs. 2c and 2d).

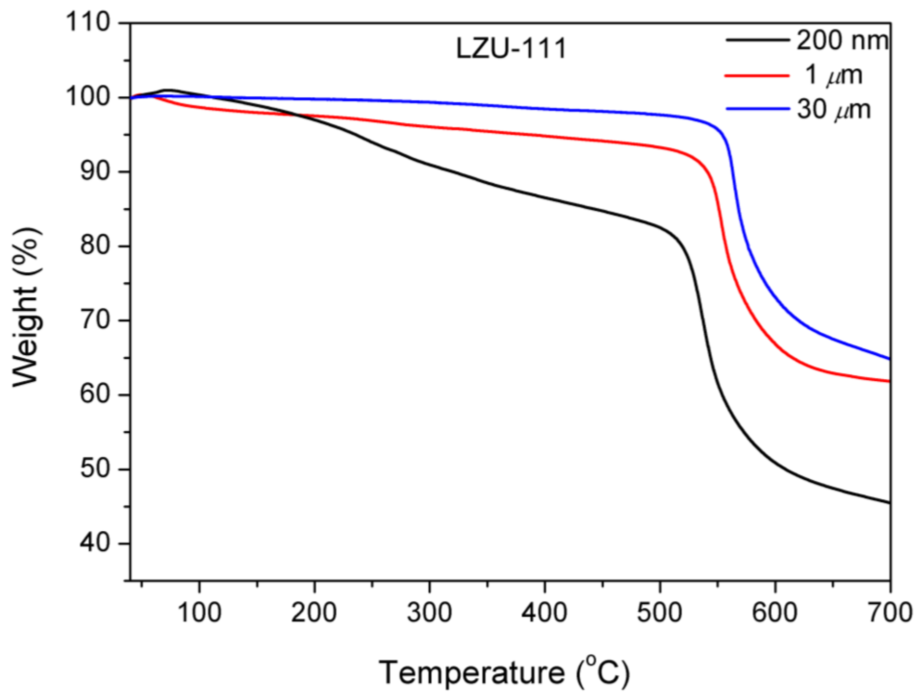

Supplementary Fig. 10 TG curves of different-sized LZU-111 (200 nm-sized, black; 1 μm-sized, red; 30 μm-sized, blue). The decomposition temperature of LZU-111 is of 547 °C for the 200 nm- and 1 μm-sized samples, and of 556 °C for the 30 μm-sized single-crystal samples. These results indicate that the thermal stability of LZU-111 is increased along with the increasing crystallinity. Before decomposition, the 200 nm- and 1 μm-sized crystals have already lost mass of 15.12% and 10.01%, respectively; while the 30 μm-sized single crystals lost mass of only 6.83% because of the less existence of the terminal defects, mosaics, or oligomers. This is consistent with the results from SSNMR and FT-IR analyses of LZU-111 (Fig. 3b and Supplementary Fig. 8).

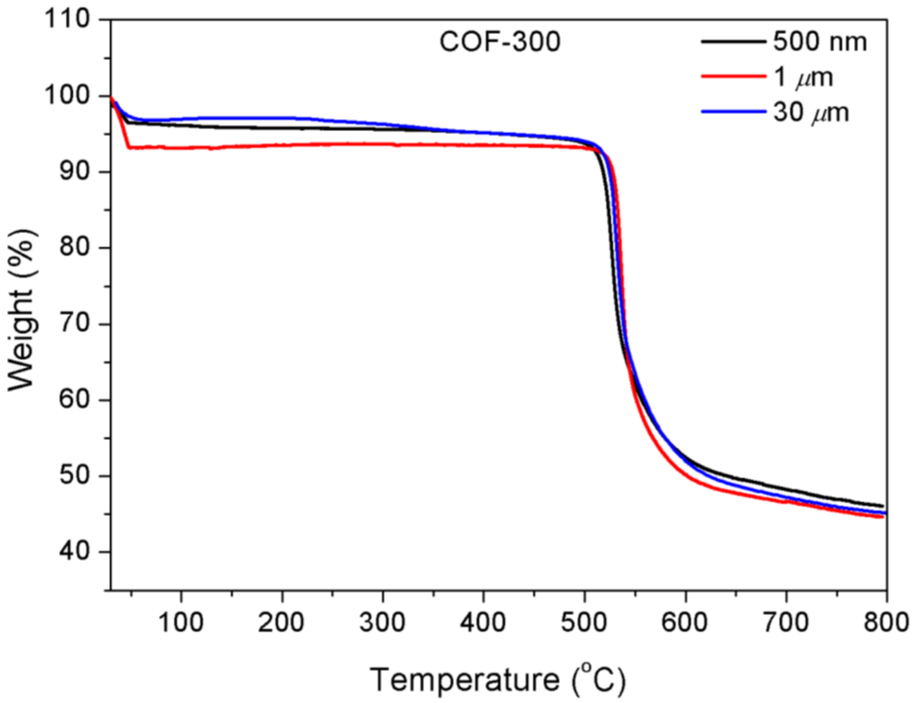

Supplementary Fig. 11 TG curves of different-sized COF-300 (500 nm-sized, black; 1 μm-sized, red; 30 μm-sized, blue). By comparing the three curves, nearly the same decomposition temperature of ~547 ºC of three-sized COF-300 samples can be observed. And before decomposition, there are few mass losses because of the less existence of the terminal defects, mosaics, or oligomers. These results indicate further that the pore integrity of three-sized COF-300 crystals are nearly the same. This is consistent with the results from PXRD, SSNMR and FT-IR analyses of COF-300.

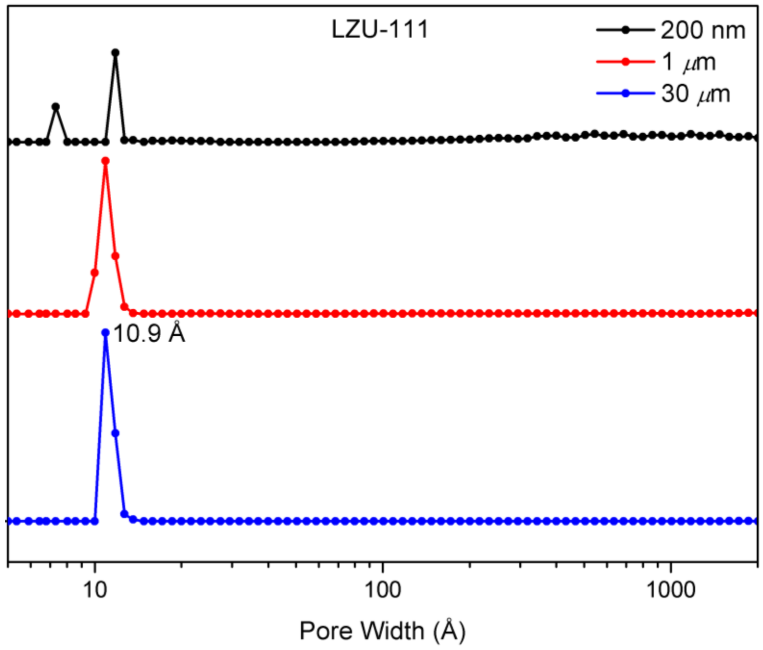

Supplementary Fig. 12 Pore size distributions (PSDs) of different-sized LZU-111 (200 nm-sized, black; 1 μm-sized, red; 30 μm-sized, blue). The PSDs were derived from N_2 adsorption data. The PSD curve is disordered in nanocrystals (centered at 7.3 Å and 11.8 Å), and meanwhile with lots of unconsolidated pores (PSD > 100 Å). On the contrary, both of 1 μm- and 30 μm-sized crystals have the PSD only centered at 10.9 Å,[4] and 30 μm-sized single-crystal samples possess a narrower PSD range, illuminating that the pores in large single crystals are arranged superior regularly. This is also in good agreement with the trends of crystallinity change (Fig. 3a) and BET surface area change (Fig. 4a).

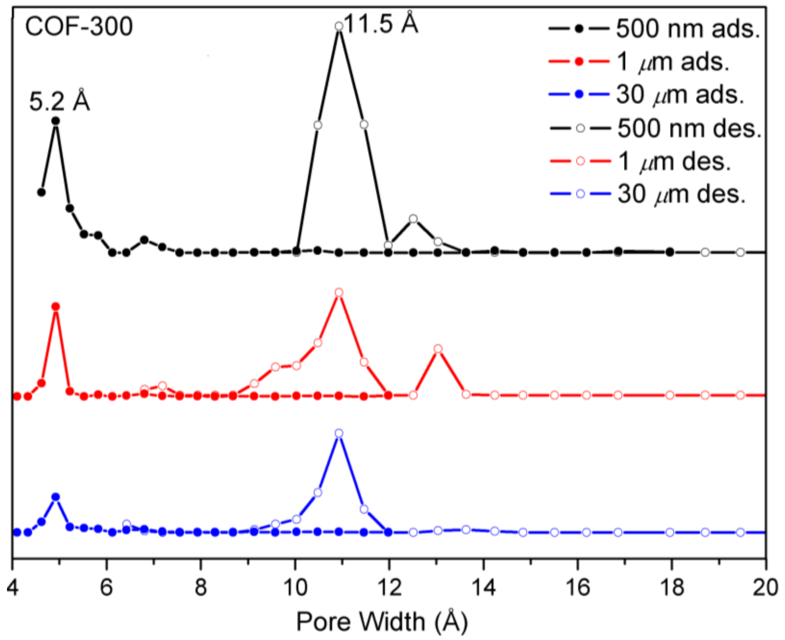

Supplementary Fig. 13 Pore size distributions of different-sized COF-300 (500 nm-sized, black; 1 μm-sized, red; 30 μm-sized, blue). The PSDs of contracted phase were derived from Ar adsorption data (solid circle), while the PSDs of expanded phase were derived from Ar desorption data (hollow circle). The contracted pore mainly centered at 5.2 Å, and the expanded pore mainly centered at 11.5 Å. The nearly identical pore size distributions prove that, the structure regularity is almost unchanged in different-sized COF-300.

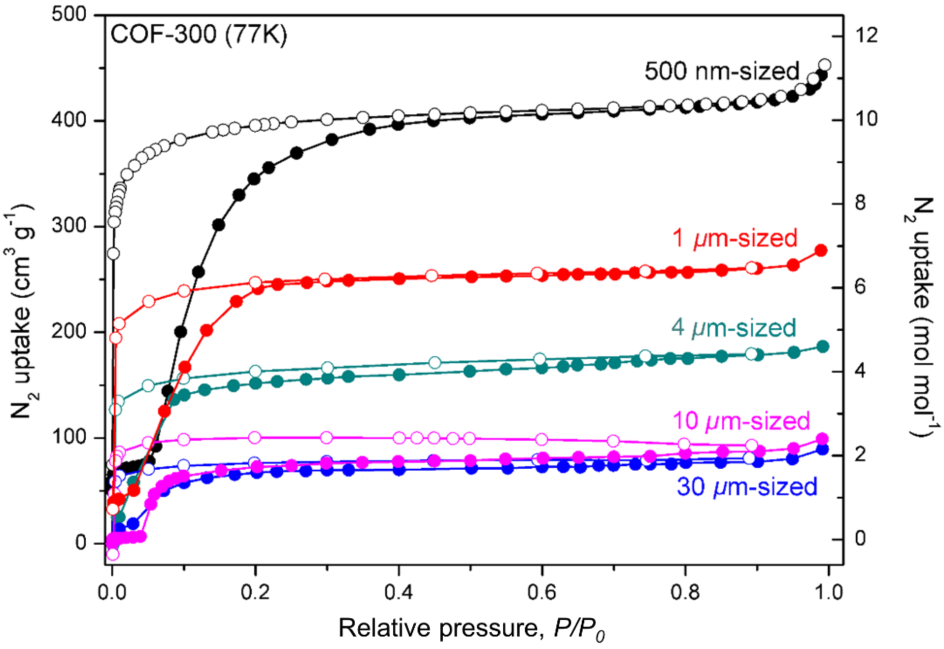

Supplementary Fig. 14 N_2 adsorption-desorption isotherms of more different-sized COF-300 (solid circle, adsorption; hollow circle, desorption. 500 nm-sized, black; 1 μm-sized, red; 4 μm-sized, turquoise; 10 μm-sized, pink; 30 μm-sized, blue). The crystal size effect influences the sorption behavior very regularly as we summarized in the main text without contingency. As shown here, N_2 sorption experiments of ~4 μm-sized and ~10 μm-sized COF-300 (see SEM images in Supplementary Fig. 5) were implemented and the isotherms were plotted and compared with those of other three sized COF-300 (500 nm-sized, 1 μm-sized, 30 μm-sized). It shows that with the crystal size increased, the overall uptakes of N_2 are decreased very regularly.

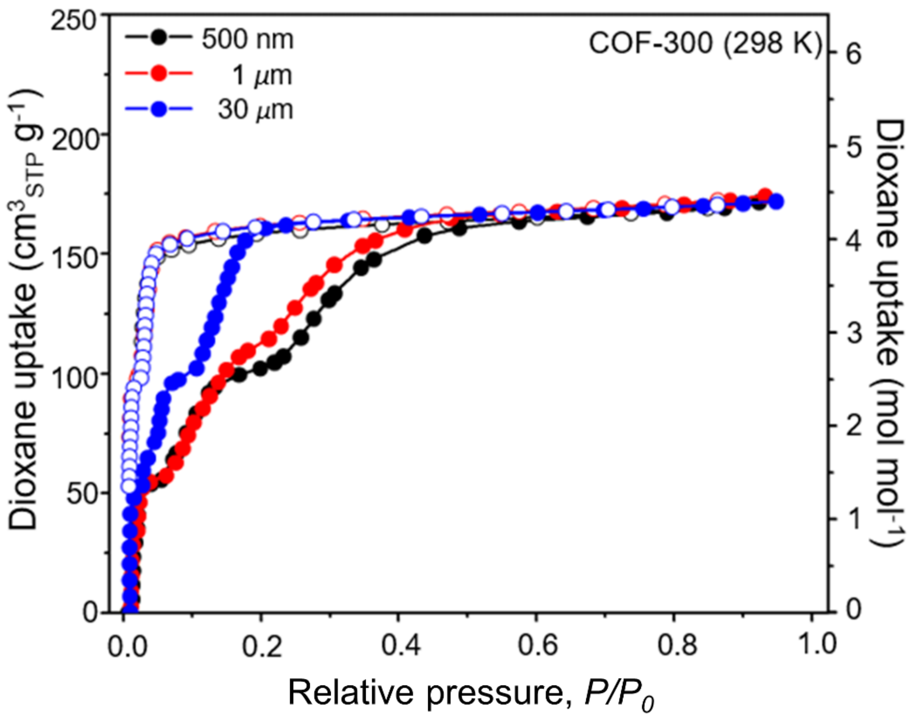

Supplementary Fig. 15 1,4-dioxane adsorption-desorption isotherms of different-sized COF-300 (solid circle, adsorption; hollow circle, desorption. 500 nm-sized, black; 1 μm-sized, red; 30 μm-sized, blue). Similar to THF adsorption (Fig. 4f), the overall 1,4-dioxane uptakes upon different-sized COF-300 are the same (~172 cm^3 g^{-1}). However, the variation tendency of 1,4-dioxane sorption in different-sized COF-300 is slightly different to that of THF sorption. In detail, not only multiple adsorption/desorption steps could be observed in more rigid 30 μm-sized COF-300, the flexible smaller crystals also have over two adsorption/desorption steps. This phenomenon maybe caused by the different host-guest interactions between COF-300 framework and 1,4-dioxane which has larger molecular size and higher polarity than THF. Even though, just like in CO_2 and THF isotherm, shorter plateaus and the steeper filling slopes can be observed in the 1,4-dioxane isotherm of 30 μm-sized crystals, due to the partially expanding mode in more rigid framework of large crystals.

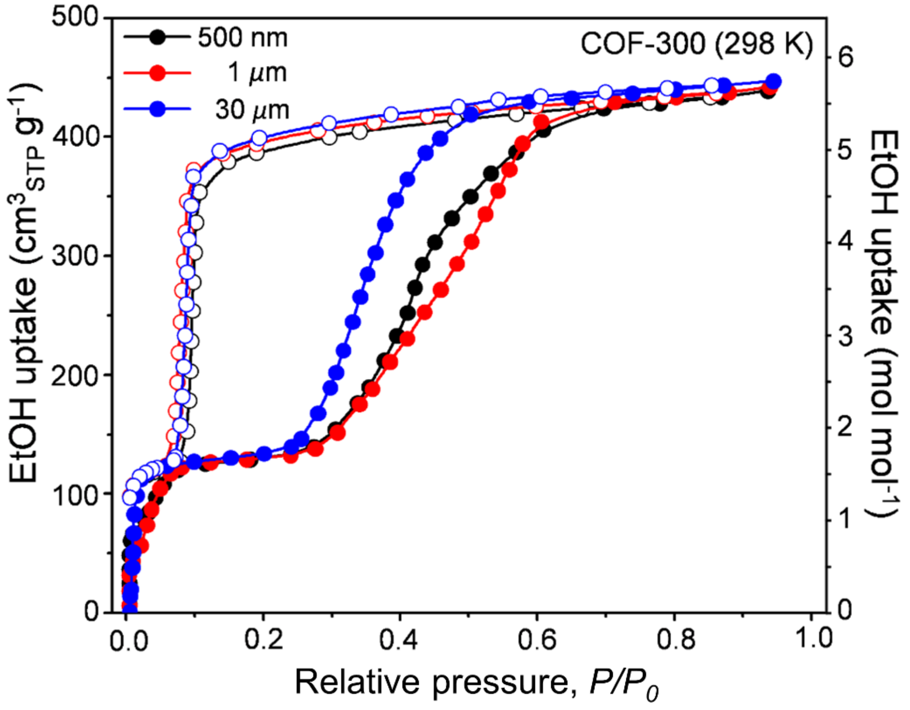

Supplementary Fig. 16 Ethyl alcohol (EtOH) adsorption-desorption isotherms of different-sized COF-300 (solid circle, adsorption; hollow circle, desorption. 500 nm-sized, black; 1 *μ*m-sized, red; 30 *μ*m-sized, blue). Similar to THF and 1,4-dioxane adsorption, the overall uptakes of ethyl alcohol upon different-sized COF-300 are approximatively the same (~220 cm^3 g^{-1}). But unlike THF and 1,4-dioxane isotherms which have distinct shapes for different-sized COF-300 crystals, the shapes of EtOH isotherms for different-sized COF-300 are very similar. In other words, there are no multiple adsorption/desorption steps with short plateaus in EtOH isotherm of COF-300 large crystals. Instead, the classical flexible-robust sorption process[6] with two obvious adsorption/desorption steps could be found in all EtOH isotherms of different-sized COF-300 crystals. It is most likely because EtOH interacts with imine bonds in COF framework via stronger H-bonds than Van der Waals' force that THF and 1,4-dioxane have. The stronger interaction makes it easier to decrease the higher energy barrier of phase transition in large crystals without going through multiple intermediate steps, which results in similar phase transition process in different-sized COF-300. However, their size effect still can be seen in different slopes. However, the rising slope of 1 *μ*m-sized COF-300 at the second

sorption stage is not between nano crystals and 30 μm-sized single-crystals, but lower than nano crystals. The possible reason is, comparing with 500 nm-sized crystals synthesized without modulator, 1 μm-sized crystals synthesized with aniline has a considerable number of different imine bonds from aniline reacting with aldehyde linkers at the edges of crystals. And due to the relatively smaller crystal size, 1 μm-sized crystals have relatively more edges than 30 μm-sized large crystal, which might induce an abnormal sorption. This is also matched with the ^{129}Xe NMR results that 1 μm-sized crystal has a shoulder peak (Supplementary Fig. 21).

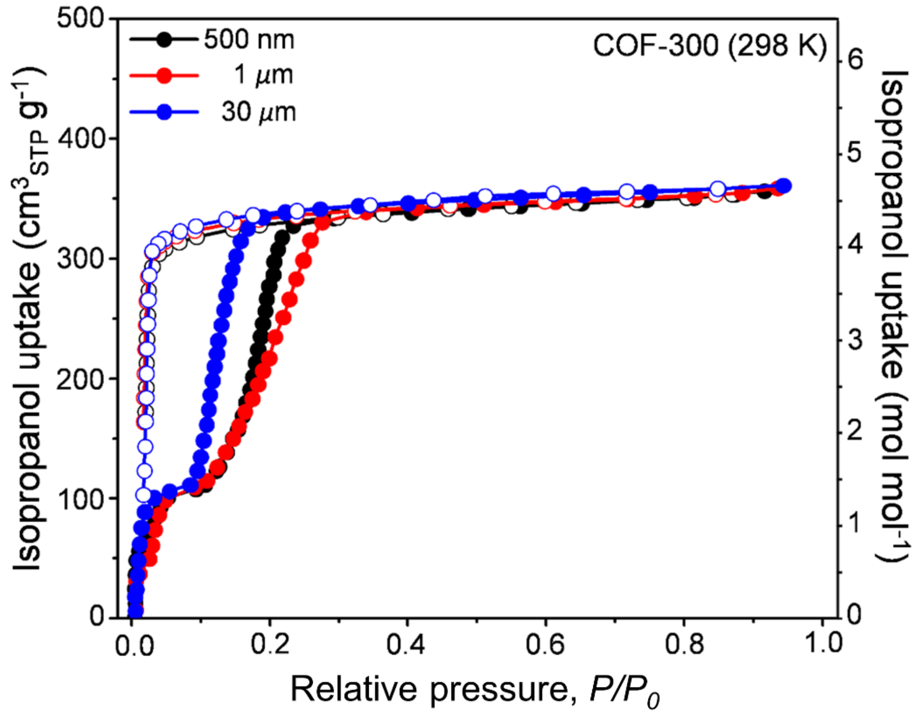

Supplementary Fig. 17 Isopropanol adsorption-desorption isotherms of different-sized COF-300 (solid circle, adsorption; hollow circle, desorption. 500 nm-sized, black; 1 *μ*m-sized, red; 30 *μ*m-sized, blue). A same variation tendency as ethyl alcohol sorption is observed in isopropanol adsorption-desorption for different-sized COF-300, verifying that more polar alcohols indeed interact with different-sized COF-300 in a different way compared with 1,4-dioxane and THF.

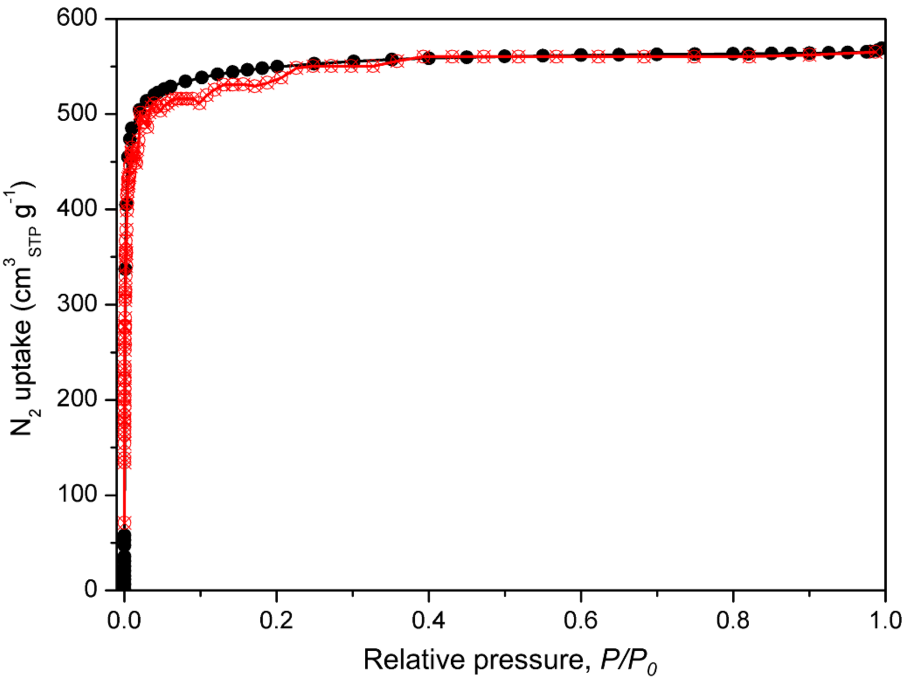

Supplementary Fig. 18 Calculated (red) and experimental (black) N_2 adsorption isotherms of LZU-111. The theoretical isotherm was calculated by the Sorption module in Material Studio 6.0 package based on the single-crystal structure of LZU-111[4]. The calculated adsorption isotherm matches very well with the experimental one. The theoretical Brunauer-Emmett-Teller (BET) surface area deduced from the calculated isotherm is 2209 m^2g^{-1} by Quantachrome NOVAWin2 v2.1 software. This data almost equal to the experimental BET surface area of 30 μm-sized crystals (2120 m^2 g^{-1}) but twice of that for 200 nm-sized polycrystals (1077 m^2 g^{-1}). All these results illuminate that 30 μm-sized single crystals are approximate perfect crystals with highly integrated channels which provide fully accessible surface area for gas adsorption.

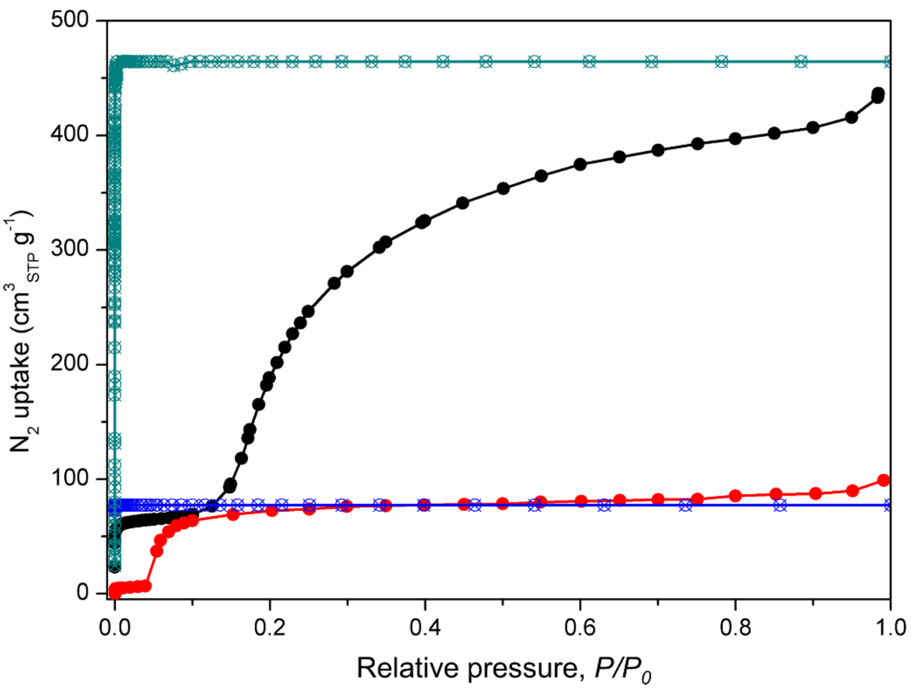

Supplementary Fig. 19 Calculated and experimental N_2 adsorption isotherms of COF-300. (Experimental N_2 isotherm of 30 μm-sized single crystals of COF-300, red; calculated N_2 isotherm of COF-300 with contracted structure, blue; experimental N_2 isotherm of 500 nm-sized crystals of COF-300, black; calculated N_2 isotherm of COF-300 with expanded structure, turquoise.) The theoretical isotherms were calculated by the Sorption module in Material Studio 7.0 package based on the contracted and expanded structural models of COF-300[4,5]. The structure parameters used in calculation are: $a = b = 19.64$ Å, $c = 8.90$ Å, $\alpha = \beta = \gamma = 90°$ with space group of $I4_1/a$ for contracted structure, $a = b = 26.22$ Å, $c = 7.57$ Å, $\alpha = \beta = \gamma = 90°$ with space group of $I4_1/a$ for expanded structure. 1) The blue isotherm matches well with the red isotherm, which means that 30 μm-sized single crystals of COF-300 keeps its contracted phase during N_2 adsorption. This result is in accordance with our conclusion in main text that inert gases only filled in the small contracted pores of rigid 30 μm-sized COF-300 and did not expand them to the large pores. 2) The final equilibrium adsorbing capacity from black isotherm is nearly the same with it from turquoise isotherm, which means that 500 nm-sized single crystals are ultimately filled with N_2 to the expanded phase. This is also in accordance with our conclusion in main text that inert gases can expand the pores of flexible nano-sized COF-300. 3)

The overlap of first plateau in black and red/ blue isotherms demonstrates that, the structural transformation indeed transformed from contracted to expanded. All these results suggest that improving theoretical calculations by considering crystal sizes is needed.

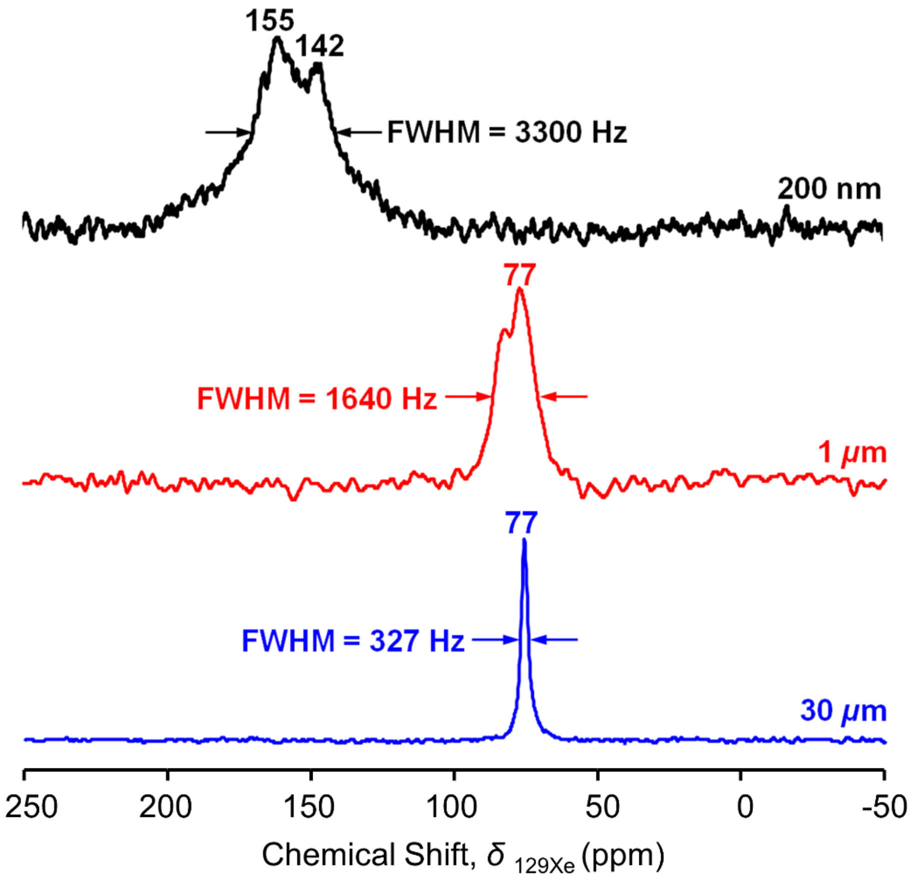

Supplementary Fig. 20 ^{129}Xe NMR spectra of xenon adsorbed within the different-sized LZU-111 (Xe@200 nm-sized LZU-111, black; Xe@1 *μ*m-sized LZU-111, red; Xe@30 *μ*m-sized LZU-111, blue). ^{129}Xe NMR was used as a unique technique for characterizing porous materials.[7,8] The FWHMs of ^{129}Xe NMR signals decrease along with the increasing crystal size of LZU-111, indicating that the regularity of the pore structure is increased along with the increasing crystal size. For example, ^{129}Xe NMR spectrum of xenon adsorbed within the 200 nm-sized crystals shows a broad and split signal with FWHM of ~3300 Hz, while the ^{129}Xe NMR spectrum for 30 *μ*m-sized single-crystal LZU-111 shows a single isotropic signal at δ = 77 ppm with a very narrow FWHM of 327 Hz, owing to the higher crystallinity with unified pores in single crystals than that in nanocrystals. The intervening FWHM of ~1640 Hz is observed for the signal of 1 *μ*m-sized LZU-111. These results are coincident with that of N_2 and Ar sorption analyses of LZU-111 (Figs. 4a–4b) that the regularity of the rigid pore structure increased with crystal upsizing. On the other

hand, the distinct chemical shifts for different-sized crystals might be related with different mean free paths of Xe diffusion, which has been exemplified in the cases of zeolites with different crystallinity.[8] Note that chemical shifts for 1 μm- and 30 μm-sized LZU-111 are very similar (~77 ppm) and largely different from that of 200 nm-sized LZU-111, which matched very well with the crystallinity change trends observed from PXRD (Fig. 3a).

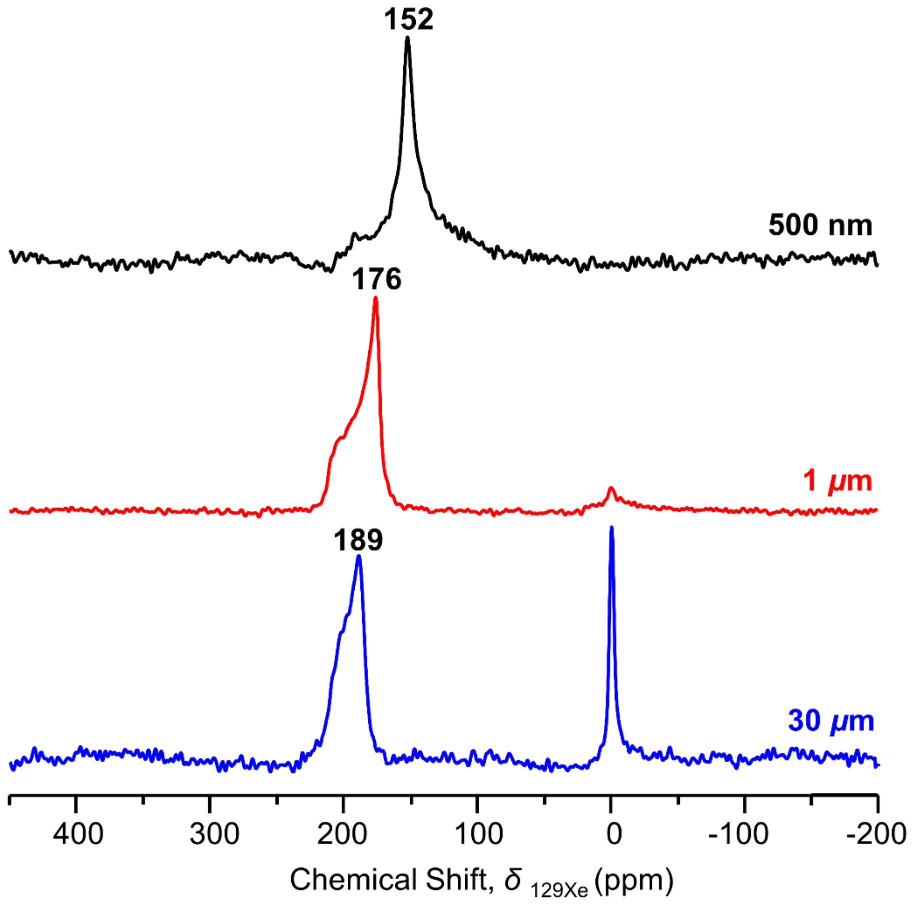

Supplementary Fig. 21 ^{129}Xe NMR spectra of xenon adsorbed within the different-sized COF-300 (Xe@500 nm-sized COF-300, black; Xe@1 μm-sized COF-300, red; Xe@30 μm-sized COF-300, blue). The chemical shifts of ^{129}Xe NMR signals increase from 152 to 176 to 189 ppm along with the increasing crystal size of COF-300, indicating that the enlarged pore size is decreased[7,8] along with the increasing crystal size. The symmetric peak shape of 500 nm-sized COF-300 implies that the pores in nano-sized COF were totally opened to uniform expanded pore, while the unsymmetrical peak shape of micro-sized COF-300 suggests that pores in these samples were partially expanded, resulting in heterogeneous pore size. Besides, as one of inert gases, the overall uptake of Xe in different sized COF-300 decrease along with the increasing crystal size because of enhanced framework rigidity, similar with sorption behaviors of N_2 and Ar in different-sized COF-300 (Figs. 4c–4d). Specifically, in the process of sample preparation of Xe adsorbing into samples before NMR, the highest sorption pressure for 30 μm-sized COF-300 is 5 mbar, while for

500 nm-sized and 1 μm-sized COF-300 are both 10 mbar. Then the three-sized Xe@COF-300 were used to collect ^{129}Xe NMR spectra. As shown in Fig. S21, there is a very high free state/gas Xe signal of 0 ppm in the spectrum of 30 μm-sized COF-300, which means that less than 5 mbar Xe can be adsorbed in the most rigid 30 μm-sized COF-300. In other word, 30 μm-sized COF-300 only absorbed part of 5 mbar Xe in the pore and the rest of Xe is still as free state/gas Xe. There are both free state/gas Xe signal of 0 ppm and adsorbed state Xe signal of 176 ppm in the spectrum of 1 μm-sized COF-300, which means that less than 10 mbar Xe can be adsorbed in 1 μm-sized COF-300. The only one adsorbed state Xe signal of 152 ppm in the spectrum of 500 nm-sized COF-300 suggests that, 500 nm-sized COF-300 absorbed all 10 mbar Xe as adsorbed state Xe. All these results verify again about our conclusion that the inert gases can hardly open the framework of 30 μm-sized COF-300 because crystal size controls structural flexibility of COF-300 by altering the number of repeating units, which eventually changes sorption selectivity.

Supplementary Table 1 Summary for controllable synthesis of different-sized LZU-111.

LZU-111							
Crystal Size	Aniline	Frozen	RT Aging	Warming (40 °C)	Heating (120 °C)	Batch 1	Batch 2
~200 nm	–	liquid N_2	0.5 h	–	3d	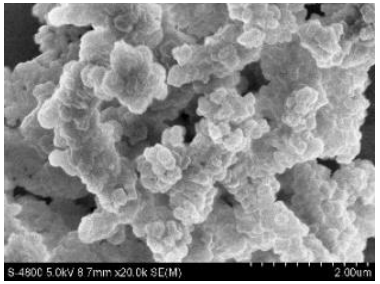	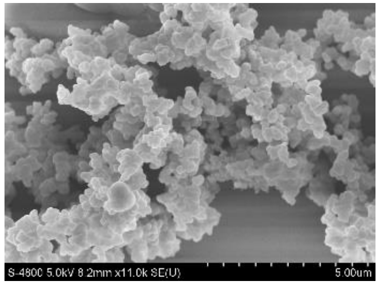
~1 μm[a]	15 eq, 0.07 mL	Liquid N_2	1d	3d	3d	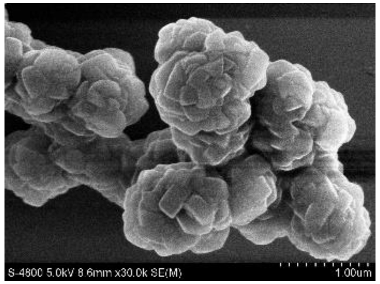	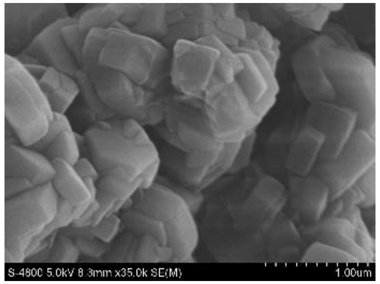
~30 μm	60 eq, 0.27 mL	Ice bath	3d	3d	4d	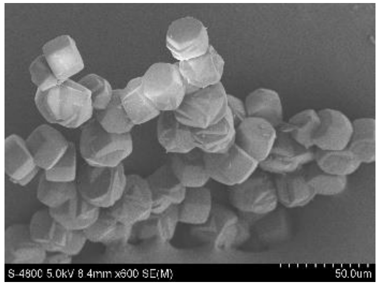	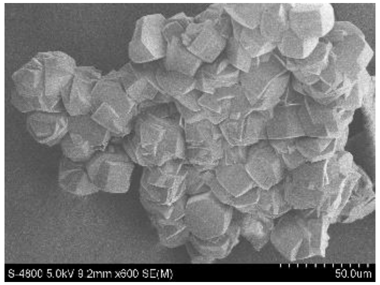

a. The crystal size distributed from 700 nm to 1 μm, represented by ~1 μm.

Supplementary Table 2 Summary for controllable synthesis of different-sized COF-300.

COF-300[a]							
Crystal Size	Aniline	Frozen	RT Aging	Warming (40 °C)	Heating (120 °C)	Sample synthesized in LZU	Sample synthesized in PKU
~500 nm	–	Liquid N_2	0.5 h	–	3d	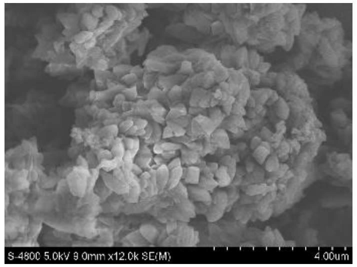	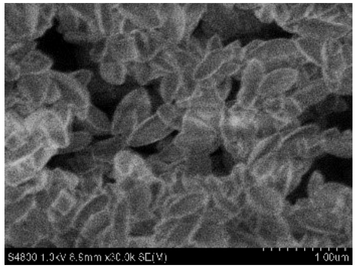
~1 μm[b]	0.6 eq,<br>5 μL	Liquid N_2	1d	3d	3d	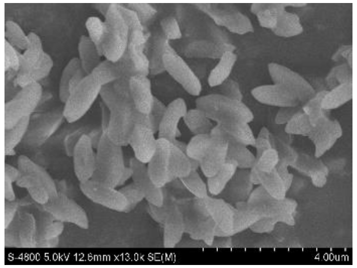	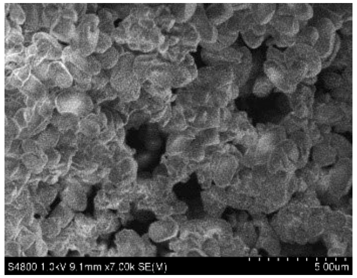
~30 μm	15 eq,<br>0.12 mL	Ice bath	3d	3d	4d	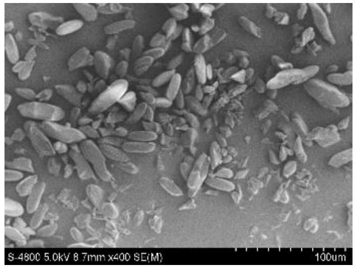	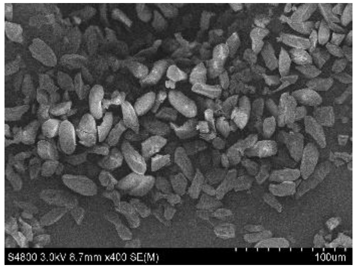

a. The samples synthesized in Lanzhou University (LZU) were operated in sealed glass tubes, and samples synthesized in Peking University (PKU) were operated in sealed pressure tubes.

b. These nanocrystals were synthesized with 15 M HOAc[5].

Supplementary Table 3 Relationship between gases/vapors properties and sorption behaviors of different sized COF-300.

Gas/ vapor	Molecular size and shape	Polarity comparison	Gate-open pressure (P/P_0)			Sorption capacity (cm^3 STP g^{-1})		
			500 nm	1 μm	30 μm	500 nm	1 μm	30 μm
Ar 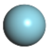	sphere	Ar< N_2<CO_2	0.05	0.025[a]	NA[b]	364.4	206.8	122.5
N_2 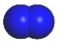	2.99 Å ellipsoid		0.05	0.028[a]	NA[b]	436.9	274.4	115.2
CO_2 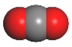	linear		0.49	0.149	0.129	372.5	423.9	426.4
Tetrahydrofuran 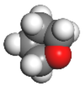	4.053 Å ring molecule	THF < 1,4-dioxane < isopropanol < ethanol	0.038	0.039	0.015	179.0	181.8	180.4
1,4-dioxane 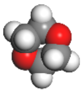	4.796 Å ring molecule		0.055	0.062	0.027	171.7	174.2	171.9
Isopropanol 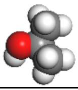	4.322 Å rod-like		0.011	0.091	0.085	178.2	179.3	180.5
Ethanol 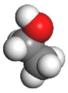	4.784 Å rod-like		0.273	0.276	0.256	219.5	221.1	223.5

a. The pores were partially opened in 1 μm-sized COF-300 with N_2 and Ar. *b*. The pores can hardly be opened in 30 μm-sized COF-300 with N_2 and Ar, see Supplementary Fig. 19.

Supplementary References

1. Duncan, N. C., Hay, B. P., Hagaman, E. W., & Custelcean, R. Thermodynamic, kinetic, and structural factors in the synthesis of imine-linked dynamic covalent frameworks. *Tetrahedron* **68**, 53–64 (2012).

2. Ganesan, P. et al. Tetrahedral n-type materials: efficient quenching of the excitation of p-type polymers in amorphous films. *J. Am. Chem. Soc.* **127**, 14530–14531 (2005).

3. Uribe-Romo, F. J. et al. A crystalline imine-linked 3-D porous covalent organic framework. *J. Am. Chem. Soc.* **131**, 4570–4571 (2009).

4. Ma, T. et al. Single-crystal x-ray diffraction structures of covalent organic frameworks. *Science* **361**, 48–52 (2018).

5. Ma, T. et al. Observation of interpenetration isomerism in covalent organic frameworks. *J. Am. Chem. Soc.* **140**, 6763–6766 (2018).

6. Li, L. et. al. Flexible-robust metal-organic framework for efficient removal of propyne from propylene. *J. Am. Chem. Soc.* **139**, 7733–7736 (2017).

7. Weiland, E., Springuel-Huet, M.-A., Nossov, A. & Gédéon, A. ^{129}Xenon NMR: review of recent insights into porous materials. *Micro. Meso. Mater.* **225**, 41–65 (2016).

8. Fraissard, J. & Ito, T. ^{129}Xe n.m.r. study of adsorbed xenon: a new method for studying zeolites and metal-zeolites. *Zeolites* **8**, 350–361 (1988).
